# Supplementary material for: A genetically encoded toolkit of functionalized nanobodies against fluorescent proteins for visualizing and manipulating intracellular signalling
Source: BMC Biol. 2019 May 23;17:41. doi: 10.1186/s12915-019-0662-4 (PMC6533734; doi:10.1186/s12915-019-0662-4)

## Slide 1
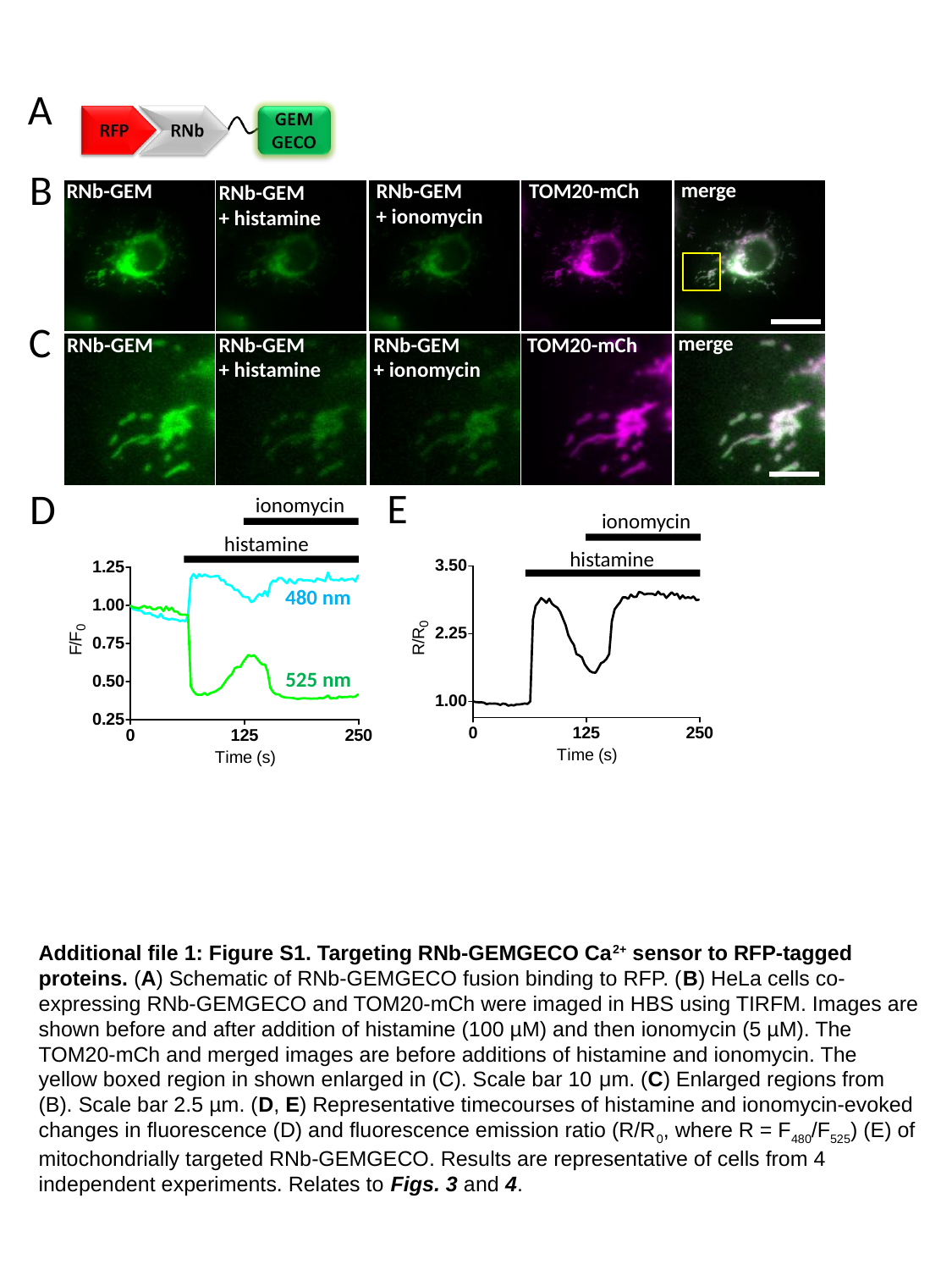

A
B
merge
RNb-GEM
+ ionomycin
TOM20-mCh
RNb-GEM
RNb-GEM
+ histamine
C
merge
RNb-GEM
+ histamine
RNb-GEM
+ ionomycin
TOM20-mCh
RNb-GEM
E
D
ionomycin
ionomycin
histamine
histamine
480 nm
525 nm
Additional file 1: Figure S1. Targeting RNb-GEMGECO Ca2+ sensor to RFP-tagged proteins. (A) Schematic of RNb-GEMGECO fusion binding to RFP. (B) HeLa cells co-expressing RNb-GEMGECO and TOM20-mCh were imaged in HBS using TIRFM. Images are shown before and after addition of histamine (100 µM) and then ionomycin (5 µM). The TOM20-mCh and merged images are before additions of histamine and ionomycin. The yellow boxed region in shown enlarged in (C). Scale bar 10 μm. (C) Enlarged regions from (B). Scale bar 2.5 µm. (D, E) Representative timecourses of histamine and ionomycin-evoked changes in fluorescence (D) and fluorescence emission ratio (R/R0, where R = F480/F525) (E) of mitochondrially targeted RNb-GEMGECO. Results are representative of cells from 4 independent experiments. Relates to Figs. 3 and 4.

## Slide 2
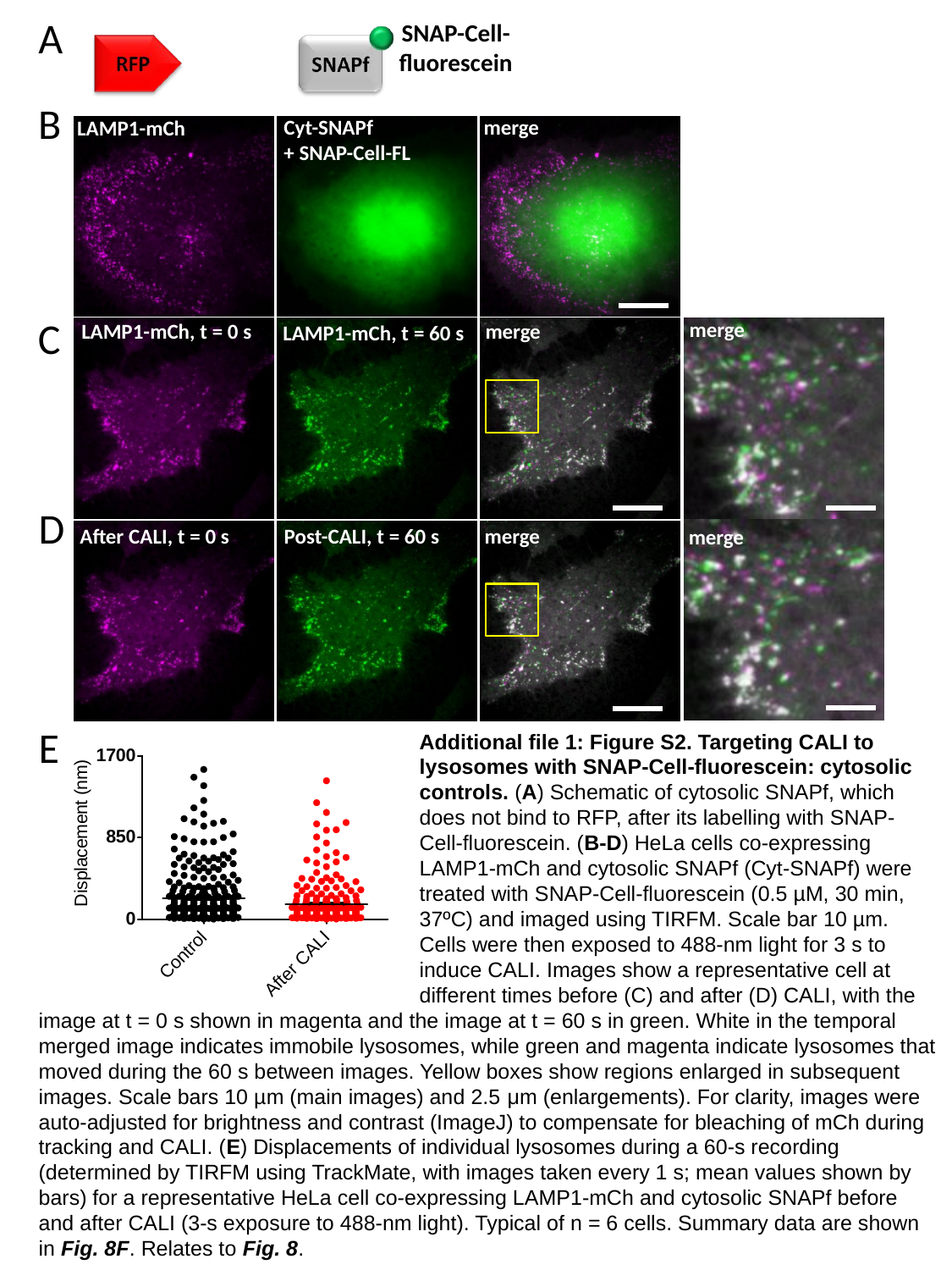

A
SNAP-Cell-fluorescein
B
Cyt-SNAPf
+ SNAP-Cell-FL
merge
LAMP1-mCh
C
merge
merge
LAMP1-mCh, t = 0 s
LAMP1-mCh, t = 60 s
D
merge
After CALI, t = 0 s
Post-CALI, t = 60 s
merge
E
			Additional file 1: Figure S2. Targeting CALI to 				lysosomes with SNAP-Cell-fluorescein: cytosolic 				controls. (A) Schematic of cytosolic SNAPf, which 				does not bind to RFP, after its labelling with SNAP-				Cell-fluorescein. (B-D) HeLa cells co-expressing 				LAMP1-mCh and cytosolic SNAPf (Cyt-SNAPf) were 				treated with SNAP-Cell-fluorescein (0.5 µM, 30 min, 				37ºC) and imaged using TIRFM. Scale bar 10 µm. 				Cells were then exposed to 488-nm light for 3 s to 				induce CALI. Images show a representative cell at 				different times before (C) and after (D) CALI, with the image at t = 0 s shown in magenta and the image at t = 60 s in green. White in the temporal merged image indicates immobile lysosomes, while green and magenta indicate lysosomes that moved during the 60 s between images. Yellow boxes show regions enlarged in subsequent images. Scale bars 10 µm (main images) and 2.5 μm (enlargements). For clarity, images were auto-adjusted for brightness and contrast (ImageJ) to compensate for bleaching of mCh during tracking and CALI. (E) Displacements of individual lysosomes during a 60-s recording (determined by TIRFM using TrackMate, with images taken every 1 s; mean values shown by bars) for a representative HeLa cell co-expressing LAMP1-mCh and cytosolic SNAPf before and after CALI (3-s exposure to 488-nm light). Typical of n = 6 cells. Summary data are shown in Fig. 8F. Relates to Fig. 8.

## Slide 3
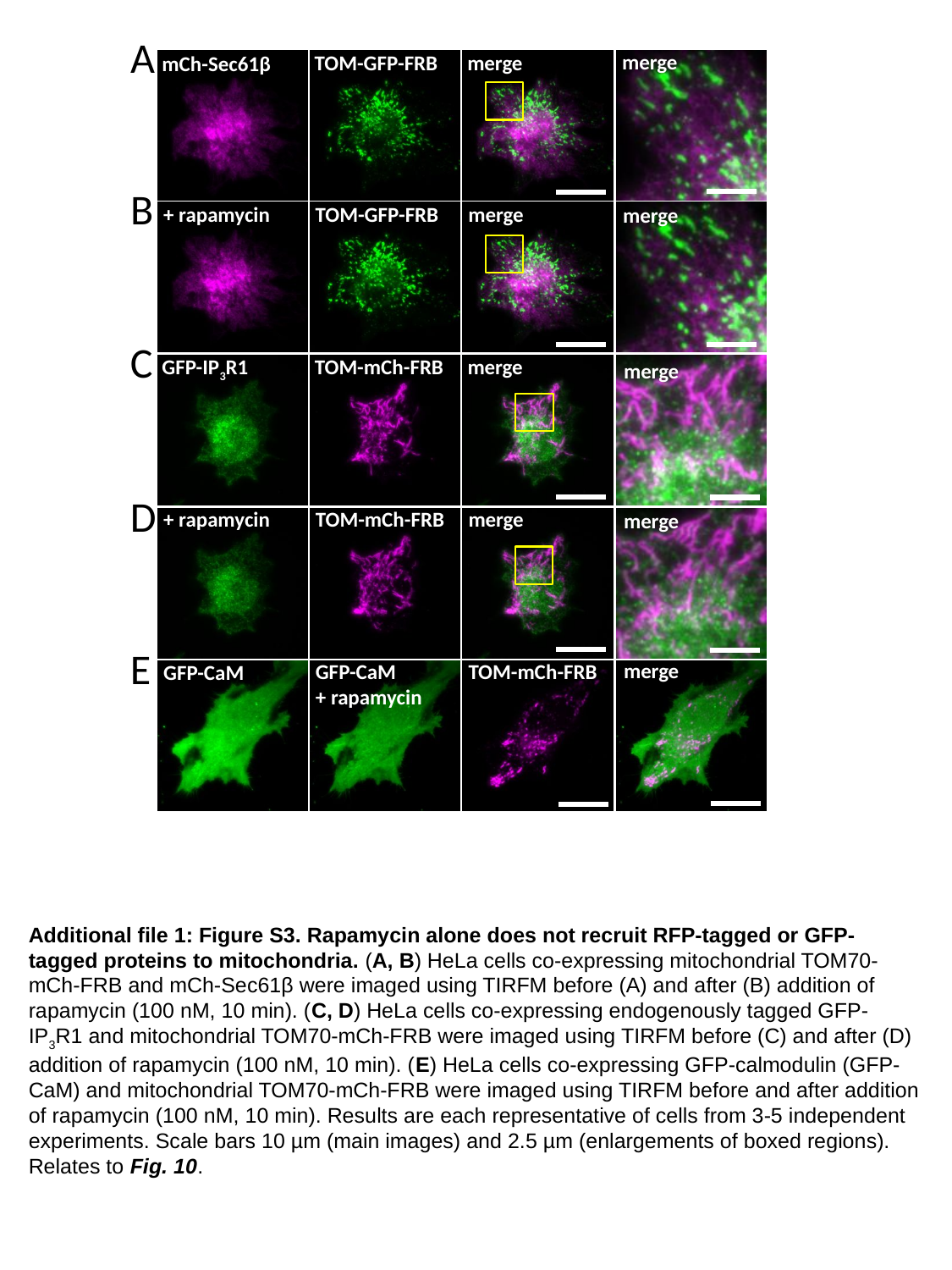

A
merge
TOM-GFP-FRB
merge
mCh-Sec61β
B
+ rapamycin
TOM-GFP-FRB
merge
merge
C
TOM-mCh-FRB
merge
GFP-IP3R1
merge
merge
D
+ rapamycin
TOM-mCh-FRB
merge
merge
E
merge
GFP-CaM
+ rapamycin
TOM-mCh-FRB
GFP-CaM
Additional file 1: Figure S3. Rapamycin alone does not recruit RFP-tagged or GFP-tagged proteins to mitochondria. (A, B) HeLa cells co-expressing mitochondrial TOM70-mCh-FRB and mCh-Sec61β were imaged using TIRFM before (A) and after (B) addition of rapamycin (100 nM, 10 min). (C, D) HeLa cells co-expressing endogenously tagged GFP-IP3R1 and mitochondrial TOM70-mCh-FRB were imaged using TIRFM before (C) and after (D) addition of rapamycin (100 nM, 10 min). (E) HeLa cells co-expressing GFP-calmodulin (GFP-CaM) and mitochondrial TOM70-mCh-FRB were imaged using TIRFM before and after addition of rapamycin (100 nM, 10 min). Results are each representative of cells from 3-5 independent experiments. Scale bars 10 µm (main images) and 2.5 µm (enlargements of boxed regions). Relates to Fig. 10.

## Slide 4
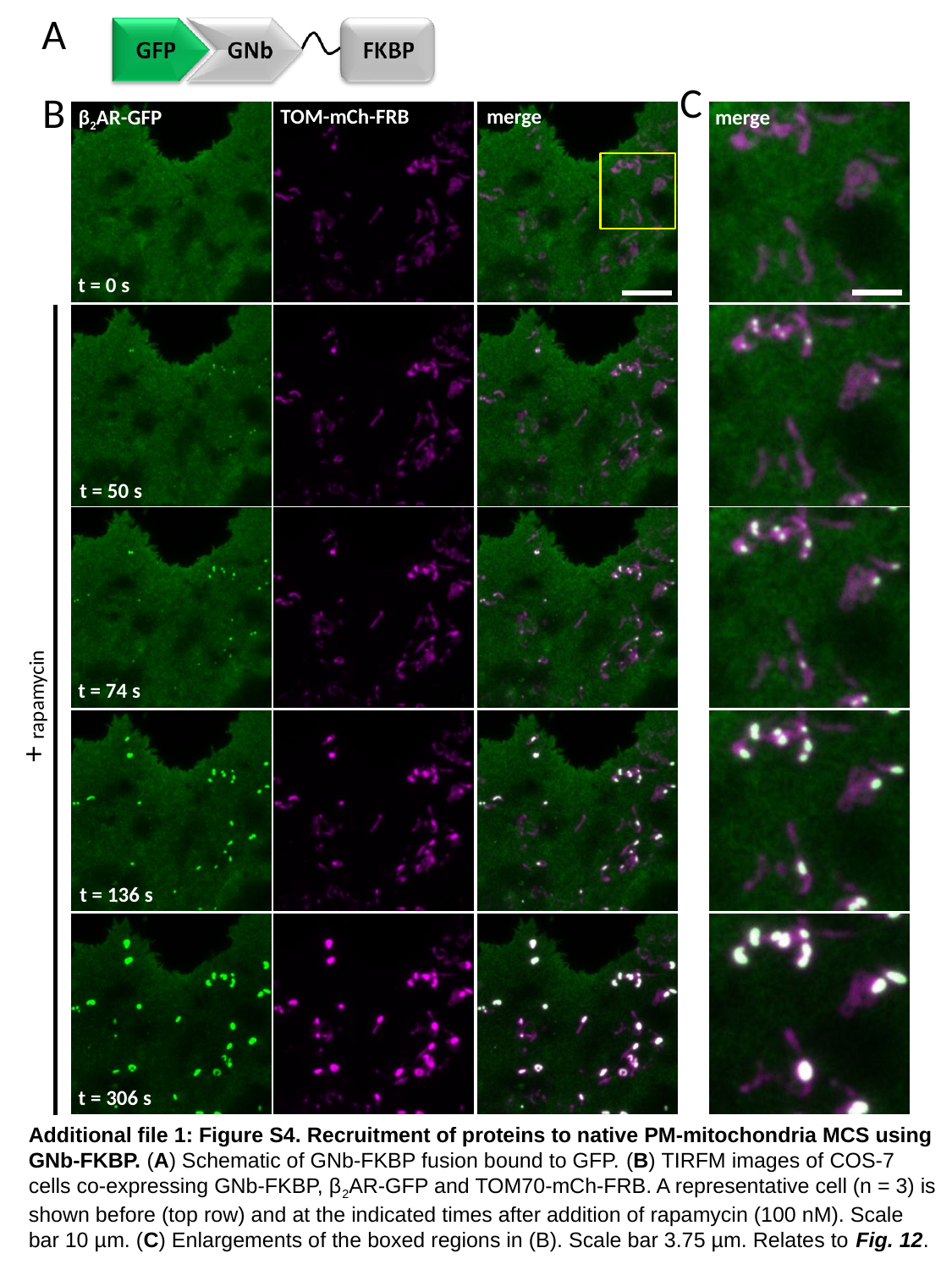

A
C
B
TOM-mCh-FRB
merge
β2AR-GFP
merge
t = 0 s
t = 50 s
+ rapamycin
t = 74 s
t = 136 s
t = 306 s
Additional file 1: Figure S4. Recruitment of proteins to native PM-mitochondria MCS using GNb-FKBP. (A) Schematic of GNb-FKBP fusion bound to GFP. (B) TIRFM images of COS-7 cells co-expressing GNb-FKBP, β2AR-GFP and TOM70-mCh-FRB. A representative cell (n = 3) is shown before (top row) and at the indicated times after addition of rapamycin (100 nM). Scale bar 10 µm. (C) Enlargements of the boxed regions in (B). Scale bar 3.75 µm. Relates to Fig. 12.

## Slide 5
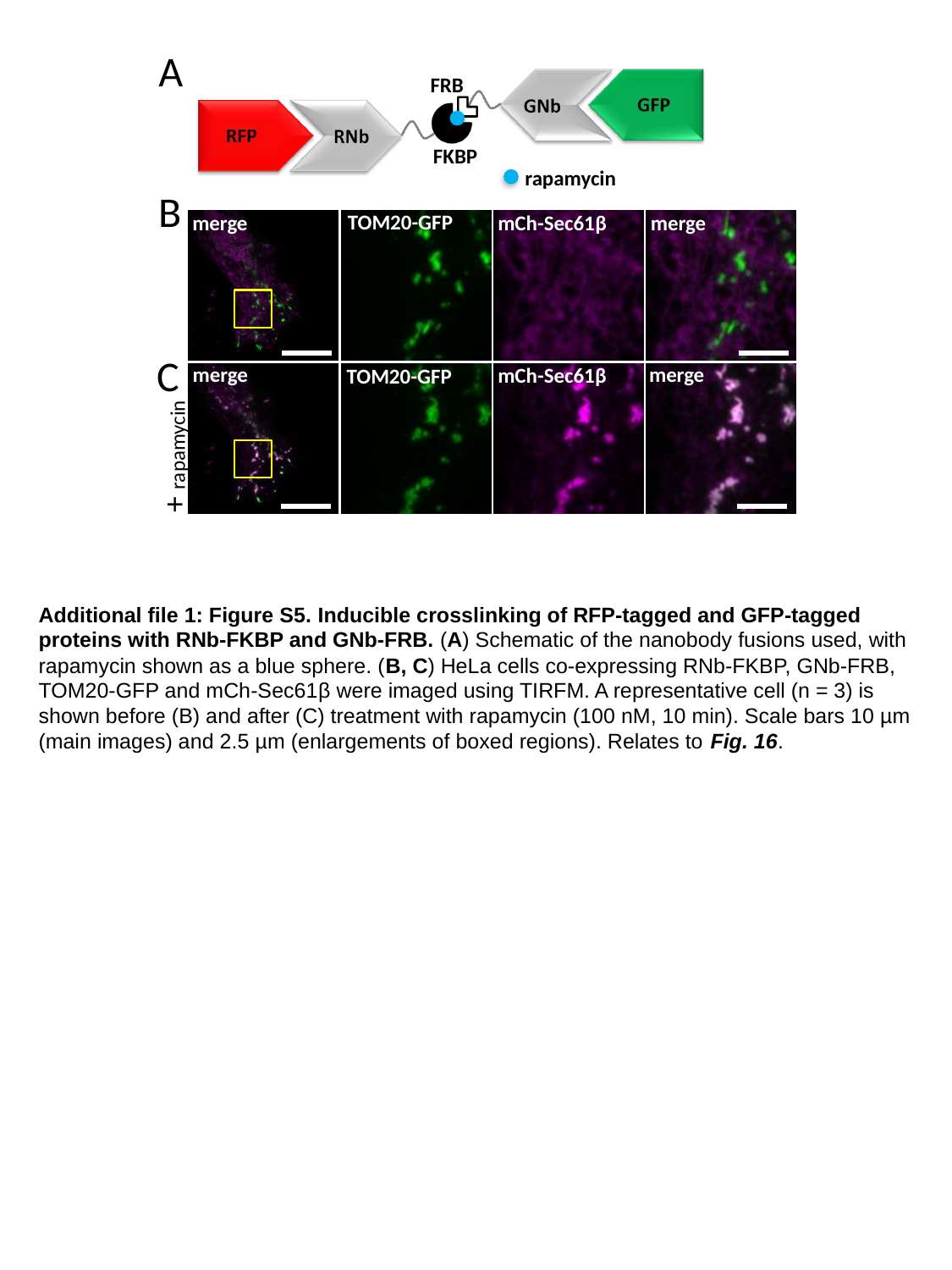

A
FRB
FKBP
rapamycin
B
TOM20-GFP
merge
merge
mCh-Sec61β
C
merge
merge
mCh-Sec61β
TOM20-GFP
+ rapamycin
Additional file 1: Figure S5. Inducible crosslinking of RFP-tagged and GFP-tagged proteins with RNb-FKBP and GNb-FRB. (A) Schematic of the nanobody fusions used, with rapamycin shown as a blue sphere. (B, C) HeLa cells co-expressing RNb-FKBP, GNb-FRB, TOM20-GFP and mCh-Sec61β were imaged using TIRFM. A representative cell (n = 3) is shown before (B) and after (C) treatment with rapamycin (100 nM, 10 min). Scale bars 10 µm (main images) and 2.5 µm (enlargements of boxed regions). Relates to Fig. 16.

## Slide 6
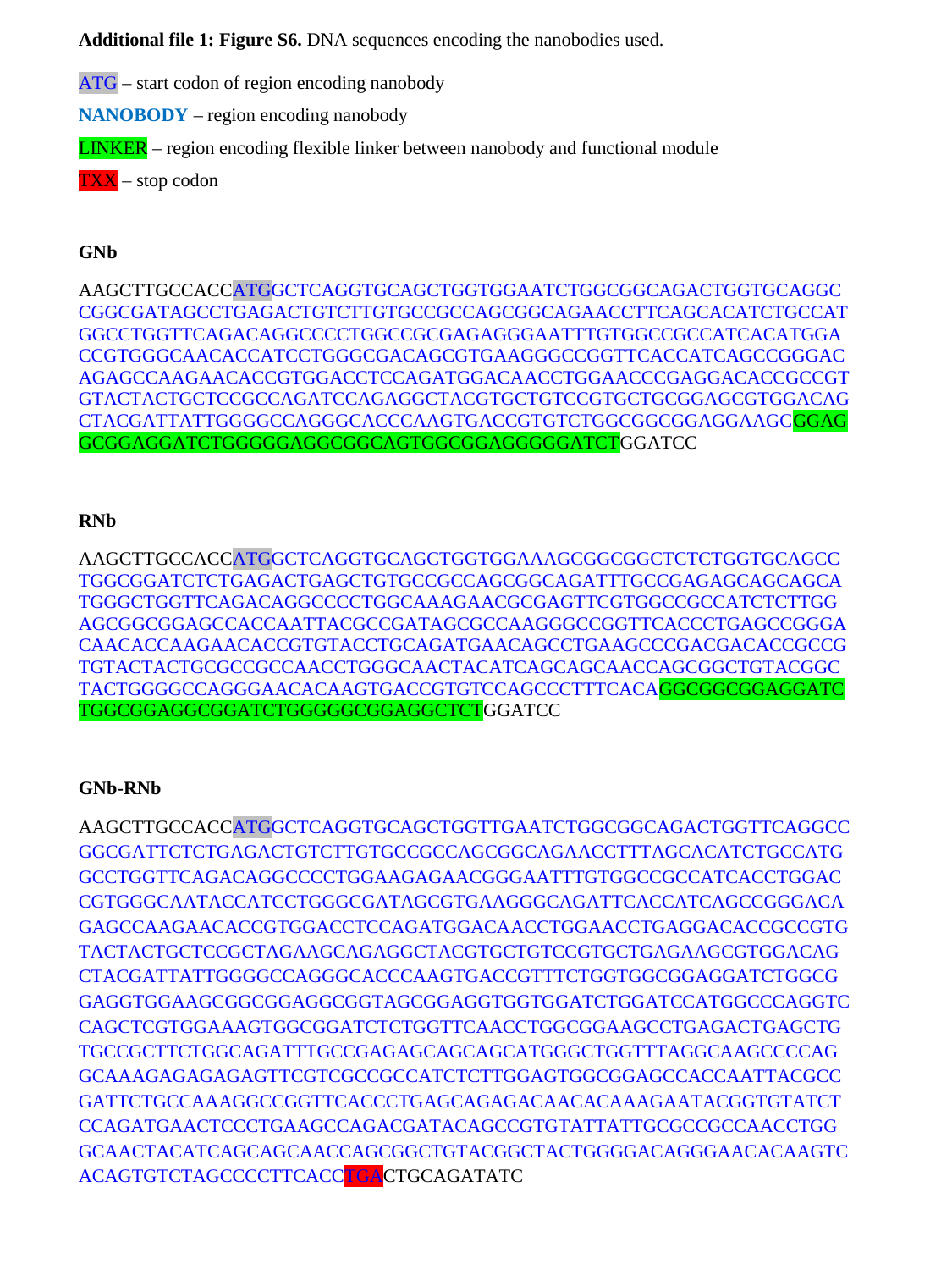

Supplement: Supplementary file 7 — Figure S1. Targeting RNb-GEMGECO Ca2+ sensor to RFP-tagged proteins. Figure S2. Targeting CALI to lysosomes with SNAP-Cell-fluorescein: cytosolic controls. Figure S3. Rapamycin alone does not recruit RFP-tagged or GFP-tagged proteins to mitochondria. Figure S4. Recruitment of proteins to native PM-mitochondria MCS using GNb-FKBP. Figure S5. Inducible crosslinking of RFP-tagged and GFP-tagged proteins with RNb-FKBP and GNb-FRB. Figure S6. DNA sequences encoding the nanobodies used. (PPTX 7375 kb) [file 12915_2019_662_MOESM1_ESM.pptx]
